# Transcriptome Sequencing Identified Genes and Gene Ontologies Associated with Early Freezing Tolerance in Maize

**DOI:** 10.3389/fpls.2016.01477

**Published:** 2016-10-07

**Authors:** Zhao Li, Guanghui Hu, Xiangfeng Liu, Yao Zhou, Yu Li, Xu Zhang, Xiaohui Yuan, Qian Zhang, Deguang Yang, Tianyu Wang, Zhiwu Zhang

**Affiliations:** ^1^Agronomy College of Northeast Agricultural UniversityHarbin, China; ^2^Department of Crop and Soil Sciences, Washington State UniversityPullman, WA, USA; ^3^Institute of Maize Research, Heilongjiang Academy of Agricultural SciencesHarbin, China; ^4^Institute of Crop Science, Chinese Academy of Agricultural SciencesBeijing, China; ^5^Department of Computer Science, Wuhan University of TechnologyWuhan, China

**Keywords:** freezing stress, maize, RNA-seq, DEGs, molecular mechanism

## Abstract

Originating in a tropical climate, maize has faced great challenges as cultivation has expanded to the majority of the world's temperate zones. In these zones, frost and cold temperatures are major factors that prevent maize from reaching its full yield potential. Among 30 elite maize inbred lines adapted to northern China, we identified two lines of extreme, but opposite, freezing tolerance levels—highly tolerant and highly sensitive. During the seedling stage of these two lines, we used RNA-seq to measure changes in maize whole genome transcriptome before and after freezing treatment. In total, 19,794 genes were expressed, of which 4550 exhibited differential expression due to either treatment (before or after freezing) or line type (tolerant or sensitive). Of the 4550 differently expressed genes, 948 exhibited differential expression due to treatment within line or lines under freezing condition. Analysis of gene ontology found that these 948 genes were significantly enriched for binding functions (DNA binding, ATP binding, and metal ion binding), protein kinase activity, and peptidase activity. Based on their enrichment, literature support, and significant levels of differential expression, 30 of these 948 genes were selected for quantitative real-time PCR (qRT-PCR) validation. The validation confirmed our RNA-Seq-based findings, with squared correlation coefficients of 80% and 50% in the tolerance and sensitive lines, respectively. This study provided valuable resources for further studies to enhance understanding of the molecular mechanisms underlying maize early freezing response and enable targeted breeding strategies for developing varieties with superior frost resistance to achieve yield potential.

## Introduction

Maize (*Zea mays* L.) is one of the most important food, feed, and biofuel crops worldwide. Originating from tropical regions, maize is highly sensitive to low temperatures, especially during the early seedling stage (Presterl et al., 2007). Freezing (<0°C) is an extreme abiotic stress for maize (Doherty et al., 2009; Guo et al., 2013) and is the most limiting factor for early planting in temperate regions (Adamczyk and Królikowski, 1998). For example, in Northeast China, cold damage occurs, on average, every 3–4 years. Maize production losses can reach 20% in the most severe cold-weather years (Ma et al., 2003). Thus, demand is high for the development of cultivars with improved freezing tolerance for longer growth periods, so that yield potentials can be fully realized in high-latitude cultivation areas.

Significant progress has been made in understanding freezing-tolerance mechanisms in plants. Several transcription factors involved in cold acclimation have been discovered. For example, C-repeat/dehydration-responsive element Binding Factors (CBFs) have been identified as key regulators of cold response that activate the expression of cold-response genes in *Arabidopsis* (Jaglo-Ottosen et al., 1998; Kasuga et al., 1999). These factors are also known as dehydration-responsive element binding factor proteins (DREBs). Among this gene family, ZmDREB1A can enhance freezing tolerance in *Arabidopsis*. This gene was also cloned in maize (Qin et al., 2004).

ICE1, a bHLH transcription factor, was identified as an inducer of CBF expression and a key regulator of CBF genes under cold conditions. Studies have demonstrated that ICE1 can improve freezing tolerance in transgenic plants (Chinnusamy, 2003). Another example is OST1 (OPEN STOMATA 1), also known as SnRK2.6/SnRK2E. OST1 is a Ser/Thr protein kinase that controls stomatal movement under stress conditions and can be activated by abscisic acid (ABA) (Grondin et al., 2015). Recently, Ding et al. (2015) found that when OST1 is activated by cold stress, it phosphorylates ICE1. In turn, the stability and transcriptional activity of ICE1 is enhanced, leading to improved freezing tolerance in *Arabidopsis*.

Besides the well-known *CBF* pathway involved in freezing tolerance, a novel molecular pathway has also been identified. TCF1 (Tolerant to Chilling and Freezing 1), a RCC1 family protein, was shown to regulate lignin biosynthesis in response to freezing stress, acting independently of the CBF pathway (Ji et al., 2015).

In addition to studies on gene functions, responses were also investigated at protein level. First identified in polar fish, ice-binding proteins are a family of protective proteins induced by freezing temperatures that have been found in many overwintering plants, conferring anti-freezing activity (Zhang et al., 2010). Recently, transgenic plants with ice-binding proteins also showed enhanced freezing tolerance (Bredow et al., 2016). Better understanding of the freezing responses at both gene and protein levels will provide more information to help uncover freezing response mechanisms.

Although maize originated in tropical regions, genetic variation in chilling tolerance exists in maize germplasms. For example, European flint and highland tropical germplasm material are more chilling tolerant than dent material from the Corn Belt (Leipner, 2009). Their genetic basis was investigated through linkage analyses and genome-wide association studies (GWAS) to map quantitative trait locus (QTL) related to maize cold tolerance. For example, a study using genome-wide association mapping for maize cold tolerance identified 19 significant association signals (Strigens et al., 2013). Many mapped QTLs are associated with seedling vigor and basic physiological processes such as photosynthesis. However, due to the limitations of phenotypic characterization, less is known about the molecular basis for adaptation to cold environments (Fracheboud et al., 2004; Hund et al., 2004). Only recently, based on multi-year field data of different maize inbred lines' cold-sensitivity and transcriptome profiling of the lines' cold response by microarray analysis, three mechanisms responsible for chilling tolerance were identified—photosynthetic apparatus modification, cell wall properties, and developmental process (Sobkowiak et al., 2016).

The transcriptome is the overall set of transcribed regions of a genome. Previously, microarray chip hybridization experiments (gene chip) were used to explore gene expression profiles (Trzcinska-Danielewicz et al., 2009; Yang et al., 2011; An et al., 2012). Next-generation, high-throughput DNA sequencing technologies now provide powerful tools for mapping and quantifying transcriptome, creating opportunities to better understand the molecular basis of cold adaptation. For example, RNA-sequencing (RNA-seq) technology can be used to characterize the entire transcriptome and to detect areas of alternative splicing (AS) and novel transcribed regions (NTRs) (Maniatis and Tasic, 2002; Shen et al., 2012; Teoh et al., 2013).

Compared to gene chip analysis, RNA-seq analysis has many advantages such as lower background signal and higher suitability for both known transcripts and new genes. Thus, RNA-seq analysis has become an efficient tool for analyzing the transcriptomic responses of many plant species to various biotic and abiotic stresses and studying the associated molecular mechanisms (Liu et al., 2012; Zhang et al., 2012; Fu et al., 2013; Shan et al., 2013; Opitz et al., 2014; Wang et al., 2014). Recently, RNA-seq analysis was also used to identify mild freezing shock response pathways in barley (Wang et al., 2016). In maize, RNA-seq analysis has been used to study the plant's response to water deficit; differentially expressed genes and significantly enriched gene ontology (GO) terms were identified (Kakumanu et al., 2012; Opitz et al., 2014). However, little is known about the transcriptomic responses of maize to freezing stress.

Because maize seedlings are usually the most vulnerable to freezing stress, responses to cold stress at the seedling stage can be used to identify important candidate genes. In this study we focused on the seedling stage of maize and used RNA-seq analysis to accomplish the following objectives: (1) exam 30 elite maize inbred lines adapted to northern China for their responses to freezing stress at seedling stage; (2) categorize genes according differentiated expression related to freezing treatment; and (3) narrow down to the potential candidate genes for future cloning studies. Increasing our understanding of the molecular mechanisms underlying freezing response in maize can enable targeted breeding strategies to accelerate the development of freezing tolerant cultivars.

## Results

### Freezing tolerance analysis

**Figure 1:**
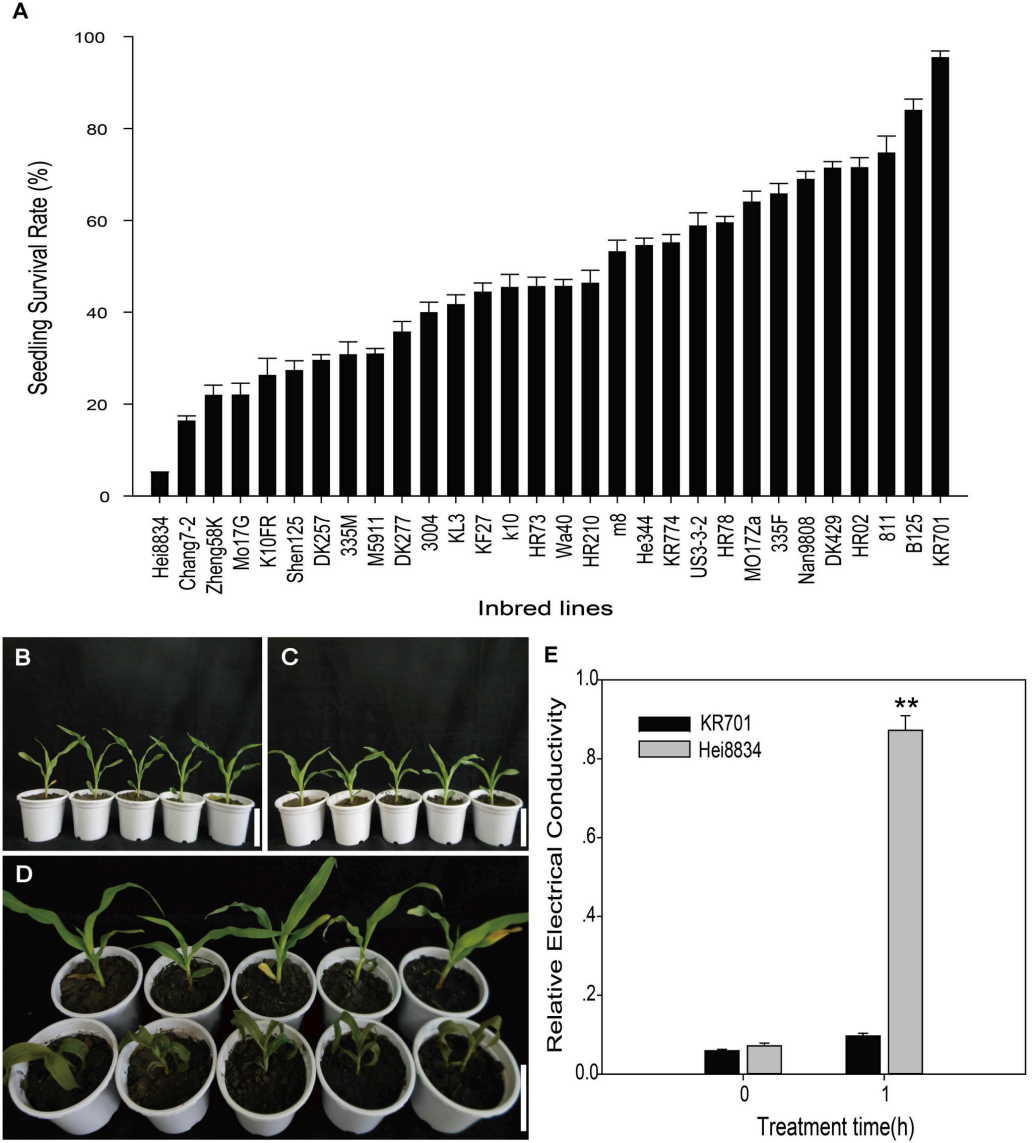
**Seedling performances under freezing treatment**. The performances were measured as survival rate and relative electrical conductivity. Survival rates were measured on the third day at 25°C after freezing treatment at −1°C for 3 h for 30 Chinese elite maize inbred lines **(A)**. Seedlings before freezing treatment are demonstrated by the two extreme lines, freezing tolerant KR701 **(B)** and freezing sensitive Hei8834 **(C)**. Visible differences were observed on the third day of recovery at 25°C after freezing treatment **(D)** between KR701 (upper row) and Hei8834 (lower row). Relative electrical conductivity was measured for KR701 and Hei8834 before freezing treatment and after 1 h of freezing treatment at −1°C **(E)**. Double asterisks indicate a statistically significant difference (*p* < 0.01) between KR701 and Hei8834. Mean values are calculated from three biological replicates; error bars represent the standard error of the mean.

A total of 30 maize inbred lines, commonly cultivated in Heilongjiang Province, China, were selected for freezing tolerance evaluation. We selected seedling survival rate as the measurement of freezing tolerance. Seedlings grown to the 3-leaf stage were subjected to a temperature of −1°C for 3 h, followed by recovery for 3 days under normal conditions (25°C). Afterward, seedling survival rates were recorded. ANOVA analysis identified significant differences between the inbred lines (Figure 1A, Table S1). Among the 30 lines, KR701 exhibited the highest seedling survival rate (97.3%), while Hei8834 exhibited the lowest (5.01%). Therefore, these two lines, KR701 (most tolerant) and Hei8834 (most sensitive), with significant difference (*p* = 5.81E-10), were selected for further analysis.

Physiological responses were recorded between these two inbred lines, including visual observations and relative electrical conductivity. The two inbred lines KR701 and Hei8834 showed little difference before freezing treatment (Figures 1B,C). After the 3-h freezing treatment (−1°C), the KR701 seedlings displayed little phenotypic change, maintaining fully expanded green leaves and intact plant architecture. In contrast, the Hei8834 seedlings showed obvious phenotypic damage, including severely wilted leaves and softened stems (Figure 1D). Relative electrical conductivity is a widely used indicator for membrane damage assay. The measurements of relative conductivity also confirmed Hei8834's severe physiological damage caused by the freezing treatment (Figure 1E).

### RNA-Seq analysis

We analyzed the transcriptomes of the freezing tolerant line (KR701, T) and freezing sensitive line (Hei8834, S) in response to freezing stress. The libraries of cDNA were prepared from these two inbred maize lines, before (Control, C) and 1 h after freezing treatment (F), and then subjected to RNA-seq profiling on the Illumina HiSeq 2000 platform. The raw data were deposited in the NCBI Sequence Read Archive (SRA) under the accession number SRX1434956. A total of about 154.4 million sequencing reads with lengths of 101 bp were generated, of which 78.84% (121.8 million reads) were mapped to the maize reference genome Zea_mays_Ensembl_AGPv3 (Table S2). Due to a technical failure, one replicate (R1) of the tolerant line after freezing stress (FT) was retrieved. All the rest three treatments (CT, CS, FS) had two replicates (R1, R2). The Q20 values of all samples, an indicator of the overall reproducibility and quality of the assay, were greater than 98% for all the replicates (Table S2). The normalized reads, referred to as fragments per kilobase of exon model per million mapped reads (FPKM), were used to estimate the total number of genes expressed in the seven samples, according to Mortazavi et al. (2008).

To analyze the similarities and differences between the seven samples, cluster analysis of all the genes using FPKM was performed. The cluster results showed clear separation between the freezing tolerant and freezing sensitive lines. And, the replicates of each treatment clustered together (Figure S1). PCA analysis of all samples also showed the same tendency as the cluster results (Figure S2). These results demonstrated that this experiment was reproducible and reliable.

### Transcriptomic responses

**Figure 2:**
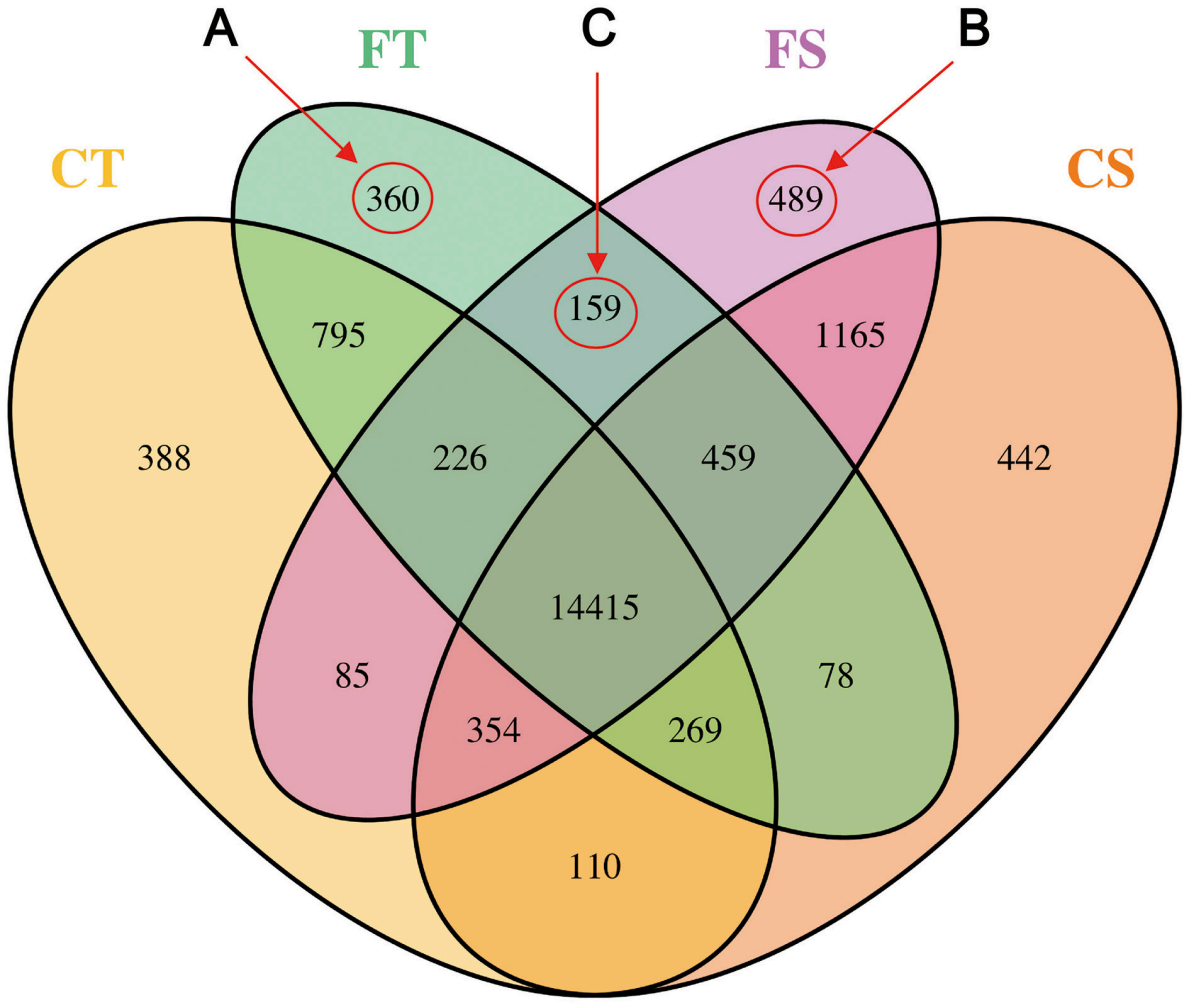
**Profile of gene expression by inbred line and freezing treatment**. The gene expression profile is illustrated as the number of transcriptomic responses by using a Venn diagram. A total of 19,794 genes were expressed. Freezing treatments are labeled Control (C) and Freezing (F). The Tolerant line (KR701) and Sensitive line (Hei8834) are labeled “T” and “S,” respectively. The biological samples of four combinations are CT, FT, CS, and FS, respectively. The area labeled “A” represents the genes specifically expressed in tolerant line KR701 after freezing treatment (FT). The area labeled “B” represents the genes specifically expressed in sensitive line Hei8834 after freezing treatment (FS). The area labeled “C” represents the freezing responsive genes shared by the tolerant and sensitive lines.

FPKM values ≥ 1 were used to determine genes expressed. A total of 19,794 annotated transcripts were identified in the four treatments with Cufflinks (Trapnell et al., 2010). The number of genes specifically expressed in each treatment, genes shared between each treatment, and genes shared among all combinations of treatments are illustrated in Figure 2. Of these transcripts, 73% (14,415) of the genes were represented in all treatments. Before freezing stress, 84% (16,642) and 87% (17,292) of the genes were expressed in the tolerant line (KR701, CT) and the sensitive line (Hei8834, CS), respectively. After freezing stress, 85% (16,761) and 88% (17,352) were expressed in KR701 (FT) and Hei8834 (FS), respectively (Table S3).

A total of 360 genes (Group A in Figure 2) were specifically expressed in tolerant line KR701 after freezing treatment (FT). GO annotation analysis (agriGO, http://bioinfo.cau.edu.cn/agriGO/analysis.php) of this group showed that GO: 0007186 (G-protein coupled receptor protein signaling pathway) and GO: 0008152 (metabolic process) were the most significantly enriched GO terms in the biological process category. Within the molecular function category, GO terms concerning nucleotide binding, ion binding, and catalytic activity were significantly enriched (Table S4).

Group B, containing 489 genes, represents the genes specifically expressed in sensitive line Hei8834 after freezing treatment (FS). In this group, GO: 0050826 (response to freezing), GO: 0045449 (regulation of transcription), and GO: 0051171 (regulation of nitrogen compound metabolic process) were the most significantly enriched GO terms in biological process. GO: 0004713 (protein tyrosine kinase activity) and GO: 0005488 (binding) were the most enriched in molecular function (Table S5).

Group C represents the expressed genes after freezing treatment that were shared by the tolerant and sensitive lines. In this group, GO: 0005488 (binding) and GO: 0004713 (protein tyrosine kinase activity) were also significantly enriched (Table S6).

### Differential expression analysis

**Figure 3:**
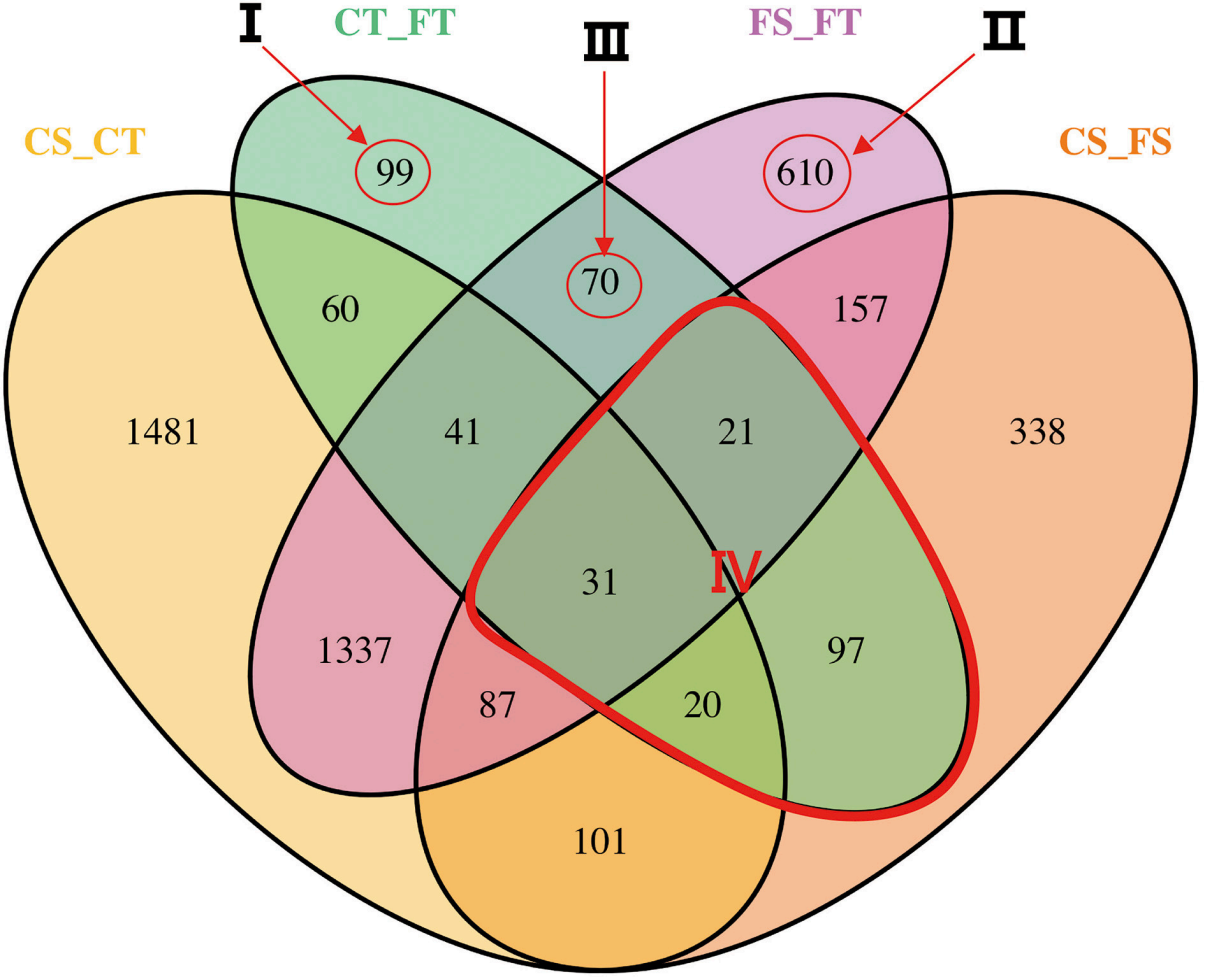
**Profile of differentially expressed genes (DEGs)**. The differentiations were compared between inbred lines under each freezing treatment, or between freezing treatments in each inbred line. Freezing treatments are labeled Control (C) and Freezing (F). The tolerant line (KR701) and sensitive line (Hei8834) are labeled “T” and “S,” respectively. The four treatment-line biological samples are control-tolerant (CT), freezing-tolerant (FT), control-sensitive (CS), and freezing-sensitive (FS). Each compared combination is separated by an underscore (e.g., CT_FT). In the Venn diagram, the numbers of DEGs are illustrated across the intersection areas among the compared combinations. In total, we found 4550 DEGs from all the areas. Some of the areas are more important than others. Four critical areas, labeled I, II, III, and IV, totally contain 948 DEGs. Area I contains the tolerant treatment response (TTR) DEGs, excluding others. Area II contains the line response under freezing (LRF) DEGs, excluding others. Area III contains both tolerance treatment response and line response under freezing (TRLR) DEGs, excluding others. Area IV contains the treatment response (TR) DEGs within line.

The software package Cuffdiff (Trapnell et al., 2012) was used to explore the differentially expressed genes (DEGs) between different treatments. Before freezing treatment, a total of 3,158 genes were differentially expressed between the tolerant and sensitive lines (see the circle labeled “CS_CT” in Figure 3). Of these DEGs, 1649 had higher expression levels in the tolerant line compared to the sensitive line (Table 1). After freezing treatment, we found 2,354 DEGs between the tolerant and sensitive lines (see the circle labeled “FS_FT” in Figure 3). Of these DEGs, 1084 had higher expression levels in the tolerant line compared to the sensitive line (Table 1). In the tolerant line, 439 genes were differentially expressed before and after freezing treatment (see the circle labeled by “CT_FT” in Figure 3); 322 of these 439 were up-regulated (Table 1). In the sensitive line, we found 852 DEGs before and after freezing treatment (see the circle labeled by “CS_FS” in Figure 3); 632 of these 852 were up-regulated (Table 1).

**Table 1:** **Number of DEGs from the four experimental comparisons**.

**Comparison**	**Total**	**Up-regulated**	**Down-regulated**
CS_CT	3158	1649	1509
CT_FT	439	322	117
CS_FS	852	632	220
FS_FT	2354	1084	1270

In total, we found 4550 DEGs among the four comparison groups, as indicated by the Venn diagram (Figure 3). The combinations of the four groups reflect the impact of lines or treatment. Some of the combinations are more important than others in aspect of freezing tolerance. Area I represents specific DEGs of CT_FT, that is, the specific freezing responsive DEGs of the freezing tolerant line. Of these 99 DEGs, 57 were up-regulated and 42 were down-regulated. Area II represents specific DEGs of FS_FT, that is, specific DEGs shared between the freezing sensitive and freezing tolerant lines after freezing treatment. Of these 610 DEGs, 244 were up-regulated and 366 were down-regulated. Area III represents the 70 specifically shared DEGs between CT_FT and FS_FT, that is, freezing responsive DEGs of the tolerant line that were also differentially expressed between the tolerant and sensitive lines after freezing treatment.

**Figure 4:**
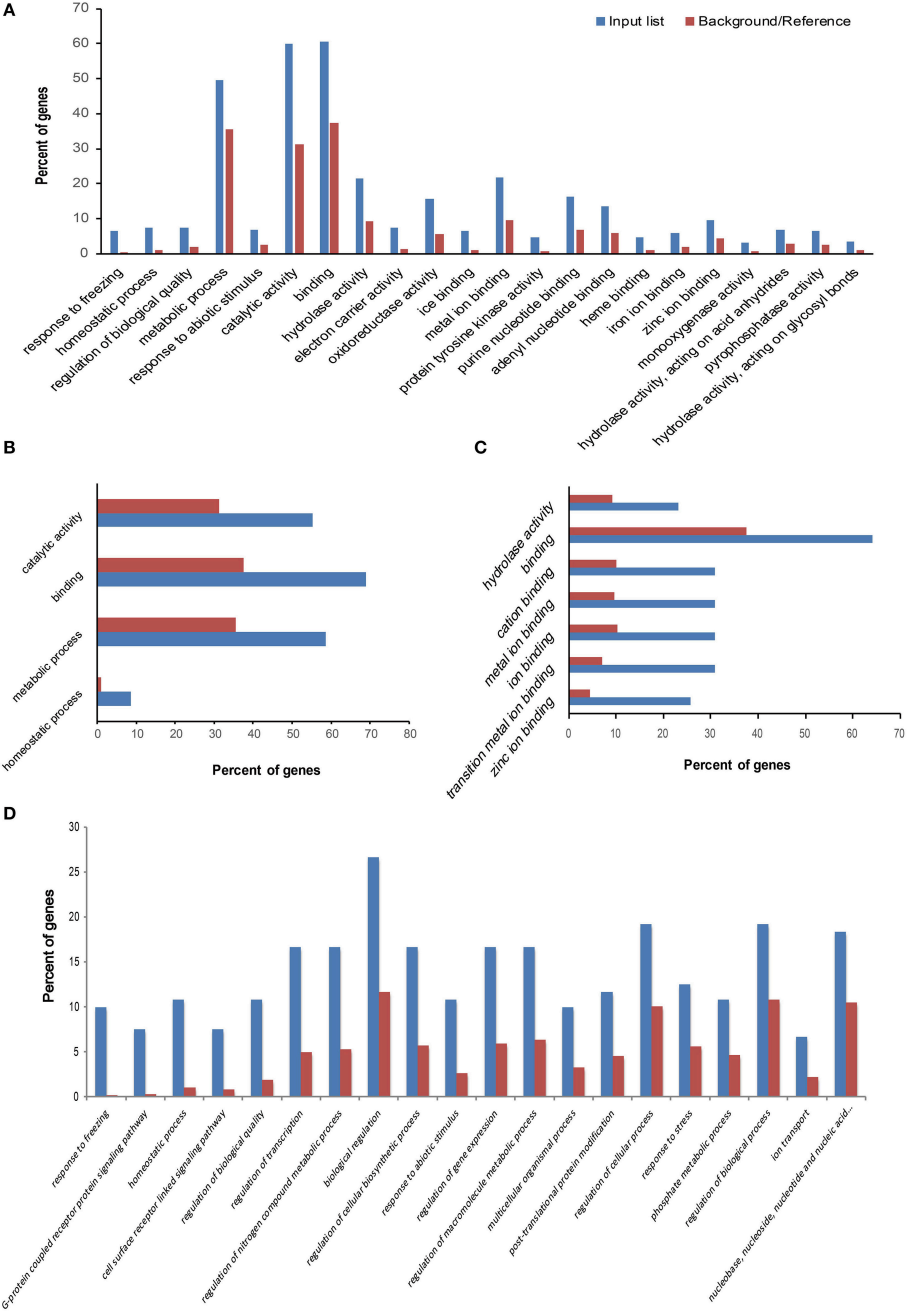
**Gene Ontology (GO) enrichment analysis of differentially expressed genes (DEGs)**. The GO analyses were performed on four sets of specific DEGs, labeled areas I, II, III, and IV in Figure 3; each area contained 99, 610, 70, and 169 DEGs, respectively. The observed gene frequency in each GO term is contrasted with the expected frequency under the null distribution (background/reference). The genes in areas II, I, III, and IV correspond to line response under freezing (LRF) **(A)**, tolerance treatment response (TTR) **(B)**, tolerance treatment response and line response under freezing (TRLR) **(C)**, and freezing treatment response in both lines (TR) **(D)**, respectively.

To investigate the biological functions of these DEGs, GO enrichment analysis was performed with agriGO (Du et al., 2010). For the 99 specific DEGs in area I (Figure 4B), GO: 0042592 (homeostatic process) and GO: 0008152 (metabolic process) were significant GO terms in biological process; GO: 0005488 (binding) and GO: 0003824 (catalytic activity) were significant GO terms in molecular function. In area II, significant GO terms in biological process included GO: 0050826 (response to freezing) and GO: 0042592 (homeostatic process), which validated our predicted biological functions for this DEG area (Figure 4A). In area III, GO terms concerning ion binding and hydrolase activity were significantly enriched (Figure 4C), suggesting DEGs with those molecular functions may contribute to the freezing response of the tolerant line and the different freezing responses between the two lines.

### Genetic difference between the tolerant and sensitive inbred lines at transcriptomic level

We anticipated that the transcriptomic differences between the tolerant and sensitive inbred lines before freezing treatment also relate the genetic difference between the two lines in responses to freezing stress. The GO enrichment analyses were performed on CT-upregulated and CS-upregulated genes of the CS_CT DEGs. The top 10 significant GO terms in biological process and molecular function were further compared (Tables 2, 3). In biological process, GO terms “metabolic process,” “response to cold,” “homeostatic process,” “response to temperature stimulus,” and “regulation of biological quality” were shared between the two lines. However, more CT-upregulated genes were enriched in these GO terms, compared to CS-upregulated genes. In CT-upregulated genes, GO terms related to metabolic process and protein phosphorylation were enriched specifically. In CS-upregulated genes, GO terms related to transcription, G-protein signaling pathway and nitrogen compound metabolic process were enriched. In molecular function, however, most of the enriched GO terms were shared. Therefore, the different number of genes in shared GO terms and the differentially enriched GO terms in biological process may provide the basis for the different freezing responses of the tolerant and sensitive inbred lines.

**Table 2:** **Enriched GO terms of CT-upregulated genes**.

**GO term**	Ontology^*^	**Description**	**Number of genes**	***p*-value**	**FDR**
GO:0008152	P	Metabolic process	474	6.70E-29	9.80E-26
GO:0009409	P	Response to cold	45	1.60E-23	1.10E-20
GO:0042592	P	Homeostatic process	54	1.60E-22	7.60E-20
GO:0009266	P	Response to temperature stimulus	45	8.60E-18	3.10E-15
GO:0044238	P	Primary metabolic process	376	5.30E-15	1.60E-12
GO:0065008	P	Regulation of biological quality	56	2.30E-13	5.60E-11
GO:0009987	P	Cellular process	413	3.60E-08	7.50E-06
GO:0044237	P	Cellular metabolic process	340	5.20E-08	9.40E-06
GO:0006468	P	Protein amino acid phosphorylation	51	5.00E-07	8.20E-05
GO:0065007	P	Biological regulation	150	1.60E-06	0.00024
GO:0003824	F	Catalytic activity	473	6.90E-43	7.30E-40
GO:0005488	F	Binding	499	2.50E-31	1.30E-28
GO:0004713	F	Protein tyrosine kinase activity	49	7.30E-26	2.60E-23
GO:0005524	F	ATP binding	123	3.00E-21	7.90E-19
GO:0032559	F	Adenyl ribonucleotide binding	123	5.10E-21	1.10E-18
GO:0032555	F	Purine ribonucleotide binding	133	2.40E-19	3.60E-17
GO:0032553	F	Ribonucleotide binding	133	2.40E-19	3.60E-17
GO:0001883	F	Purine nucleoside binding	125	9.60E-19	1.00E-16
GO:0001882	F	Nucleoside binding	125	9.60E-19	1.00E-16
GO:0030554	F	Adenyl nucleotide binding	125	9.60E-19	1.00E-16

* *Biological Process (P) and Molecular Function (F)*.

**Table 3:** **Enriched GO terms of CS-upregulated genes**.

**GO term**	Ontology^*^	**Description**	**Number of genes**	***p*-value**	**FDR**
GO:0008152	P	Metabolic process	412	2.80E-21	3.70E-18
GO:0009409	P	Response to cold	31	3.00E-14	1.60E-11
GO:0042592	P	Homeostatic process	39	3.80E-14	1.60E-11
GO:0009266	P	Response to temperature stimulus	31	2.80E-10	9.10E-08
GO:0045449	P	Regulation of transcription	84	3.80E-10	9.70E-08
GO:0006350	P	Transcription	88	8.80E-10	1.90E-07
GO:0007186	P	G-protein coupled receptor protein signaling pathway	16	2.40E-09	4.50E-07
GO:0019219	P	Regulation of nucleobase, nucleoside, nucleotide and nucleic acid metabolic process	84	3.50E-09	5.10E-07
GO:0051171	P	Regulation of nitrogen compound metabolic process	84	3.50E-09	5.10E-07
GO:0065008	P	Regulation of biological quality	41	6.80E-08	7.70E-06
GO:0005488	F	Binding	468	1.80E-34	1.60E-31
GO:0003824	F	Catalytic activity	410	2.40E-32	1.00E-29
GO:0009055	F	Electron carrier activity	59	8.70E-23	2.50E-20
GO:0032559	F	Adenyl ribonucleotide binding	116	2.20E-21	2.70E-19
GO:0001883	F	Purine nucleoside binding	123	2.20E-21	2.70E-19
GO:0001882	F	Nucleoside binding	123	2.20E-21	2.70E-19
GO:0030554	F	Adenyl nucleotide binding	123	2.20E-21	2.70E-19
GO:0004713	F	Protein tyrosine kinase activity	41	3.60E-21	3.50E-19
GO:0005524	F	ATP binding	115	3.60E-21	3.50E-19
GO:0017076	F	Purine nucleotide binding	135	5.10E-21	4.40E-19

* *Biological Process (P) and Molecular Function (F)*.

### Common freezing responsive genes

As shown in Figure 3, area IV represents the 169 DEGs shared by CT_FT and CS_FS. GO enrichment analysis for these DEGs is illustrated in Figure 4D. Of these shared genes, 151 were up-regulated in both CT_FT and CS_FS (UU genes); 13 were down-regulated in both CT_FT and CS_FS (DD genes); two were up-regulated in CT_FT, but down-regulated in CS_FS (UD genes); and three were down-regulated in CT_FT, but up-regulated in CS_FS (DU genes). We designated the 151 UU and 13 DD genes as common freezing responsive genes between the tolerant and sensitive lines. Results from the gene ontology analysis of these common freezing responsive genes are provided in Table S7. For biological process, GO terms “response to freezing,” “G-protein coupled receptor protein signaling pathway,” “homeostatic process,” and “regulation of transcription” were significantly enriched. These results suggest that these processes may participate in the response of maize seedlings to freezing conditions. For molecular function, genes which function in binding (ice binding, DNA binding, metal ion binding, etc.), catalytic activity (protein tyrosine kinase activity, hydrolase activity, phosphatase activity, etc.), and receptor activity were significantly enriched. The GO term “integral to membrane part” was the only one enriched relative to cellular component.

### Common freezing responsive genes with differential response levels

Some of the 164 common freezing responsive genes exhibited differential response levels between the tolerant and sensitive lines. We calculated the ratio of CT_FT log2 fold change to CS_FS log2 fold change. Using ratios ≥ 2 or ≤ 0.5 as selection criteria, we found a total of eight common freezing responsive genes with significant differential response levels.

With these eight genes, and the two UD genes and three DU genes, we performed a homolog search in Arabidopsis (Table S8). Previous studies report that many of the homologous *Arabidopsis* genes we identified are involved in abiotic stress response processes, including cold stress. For example, AT1G72360 (homolog of GRMZM2G148333) encodes a member of the ethylene response factor (ERF) subfamily B-2 of the ERF/AP2 transcription factor family and participates in low oxygen signaling (Licausi et al., 2010). GRMZM2G148333's homolog in rice, LOC_Os09g26420, has been identified as *OsBIERF1*, which can be induced by treatments with BTH (benzothiadiazole), salicylic acid, salt, cold, drought, and wounding. This finding suggests that *OsBIERF1* may participate in different signaling pathways that mediate both biotic and abiotic responses (Cao et al., 2006). AT1G80920 (homolog of GRMZM2G086841) encodes a chloroplast-targeted DnaJ protein, AtJ8, which is negatively regulated by light and stress (Chen et al., 2011). Additionally, AT1G80920 is down-regulated in the Arabidopsis mutant *sfr6*, which shows more freezing sensitivity after cold acclimation compared to its wild counterpart (Boyce et al., 2003). AT3G28210 (homolog of GRMZM2G125775) is also a stress-associated protein 12 (SAP12) and is strongly induced under cold and salt stress (Ströher et al., 2009). AT5G56550 (homolog of GRMZM2G076844) encodes OXIDATIVE STRESS 3 (OXS3), a putative N-acetyltransferase or thioltransferase, which participates in heavy metal and oxidative stress (Blanvillain et al., 2009). AT2G36460 (homolog of GRMZM2G057823) encodes fructose-bisphosphate aldolase 6, which is involved in response to cadmium ion, salt stress, and pentose-phosphate shunt (Lu et al., 2012).

In addition, many gene homologs participate in plant hormone signaling. For example, AT1G18350 (homolog of GRMZM2G344388), also known as ATMKK7 or BUD1, encodes a MAP kinase kinase. BUD1 is a negative regulator of polar auxin transport and its overexpression activates both basal and systemic acquired resistance (Zhang et al., 2008). AT1G76680 (homolog of GRMZM2G000236) encodes a 12-oxophytodienoic acid reductase, which is up-regulated by senescence and jasmonic acid (He and Gan, 2001). AT4G08950 (homolog of GRMZM2G149422), also known as EXORDIUM (EXO), responds to brassinosteroid stimulus (Müssig et al., 2006).

### DEGs related to “response to freezing”

Of the 17 DEGs in area II (Figure 3) that were assigned GO term “response to freezing,” nine genes had higher expression levels and eight genes had lower expression levels in the tolerant line compared to the sensitive line. Detailed information about these genes, including their homologs in Arabidopsis, can be found in Table S9.

Of the nine genes with higher expression levels in the freezing tolerant line, GRMZM2G075974 and GRMZM2G115422 encode transferase. GRMZM2G075974 encodes a glutamine amidotransferase and may be involved in defense response. AT2G39980, the Arabidopsis homolog of GRMZM2G115422, encodes a HXXXD-type acyl-transferase, which may be involved in response to karrikin (Nelson et al., 2010). Two genes encode ion-binding proteins. AT4G14040, the homolog of GRMZM2G103812, encodes a selenium-binding protein and is involved in cadmium detoxification processes (Dutilleul et al., 2008). GRMZM2G459663, which encodes a Calcium-binding EF-hand family protein, may be involved in abiotic and biotic stress (Davletova et al., 2005; Ascencio-Ibanez et al., 2008). GRMZM2G079956, which encodes a BAG family protein, was up-regulated in the freezing tolerant line. Plant BAG proteins have been reported to participate in both abiotic biotic stress (Doukhanina et al., 2006). GRMZM2G348452 encodes a cytokinin dehydrogenase and was also up-regulated in the freezing tolerant line. Cytokinin dehydrogenase catalyzes the irreversible degradation of cytokinins isopentenyladenine, zeatin, and their ribosides in a single enzymatic step by oxidative side-chain cleavage. Thus, this enzyme plays an important role in regulating cytokinin functions and participates in plant aging and senescence processes (Schmülling et al., 2003; Mýtinová et al., 2011). AT2G41475 (homolog of GRMZM2G061932), also known as ATS3, is involved in stomatal closure (Van Hove et al., 2015).

Eight genes had higher expression levels in the freezing treated sensitive line compared to the tolerant line. This finding suggests a differential response strategy or simply a response caused by different degrees of freezing damage. AT1G12520 (homolog of GRMZM2G175728) encodes a copper chaperone for SOD1 and is up-regulated in response to copper and senescence (Huang et al., 2012; Kuo et al., 2013). GRMZM2G044194 encodes phytosulfokine, which is an important small peptide signal (Yang et al., 2001). AT1G56010 (homolog of GRMZM2G114850), also known as NAC1, encodes a NAC transcription factor, which is involved in auxin signaling (Xie et al., 2000). AT1G56010's homolog in Brassica napus is involved in response to multiple biotic and abiotic stresses, such as physical wounding, pathogen infection, low temperature, and drought (Hegedus et al., 2003). GRMZM2G180335 encodes a Dynamin GTPase effector, which localizes to the chloroplast, mitochondrion, and peroxisome and is involved in peroxisome and mitochondria fission (Arimura et al., 2008; Aung and Hu, 2009). GRMZM2G178038, which encodes a Zinc finger family protein, may participate in protein degradation (Boisson et al., 2003).

### Quantitative real-time RT-PCR (qRT-PCR) confirmation

**Figure 5:**
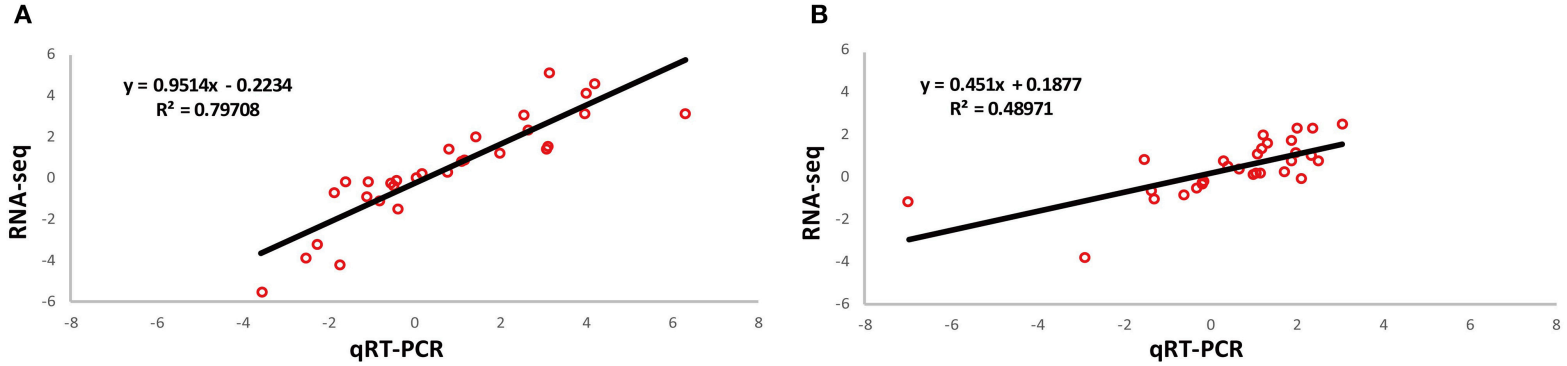
**Validation of RNA-seq expression through qRT-PCR**. Validation was performed in both freezing tolerant maize inbred line KR701 **(A)** and freezing sensitive maize inbred line Hei8834 **(B)**. The plots demonstrate the expression ratio between before and after freezing treatment in Log scale with base of two.

To confirm our findings based on the RNA-seq data, we conducted a validation experiment by using quantitative real-time PCR (qRT-PCR). We made the selection of genes based on these criteria: highly differentiated in response to freezing and reported to be potentially associated with cold resistance. In total, 30 genes were selected from the identified DEGs. Results of the qRT-PCR analysis confirmed our findings based on RNA-seq data (Table S11). The patterns of RNA-seq expression on 28 genes were replicated by the qRT-PCR approach. Only two genes (ZmGRMZM2G076844 and ZmGRMZM2G476685) showed different expression patterns in the sensitive line. Correlation of fold changes before and after freezing treatment between qRT-PCR and RNA-seq are shown in Figure 5.

## Materials and methods

### Freezing resistance evaluation of maize inbred lines

Thirty maize (*Zea mays*) inbred lines (provided by Heilongjiang Academy of Agricultural Sciences) were used for assessing seedling freezing tolerance. Seeds were surface sterilized with 75% (v/v) ethanol for 3 min, then rinsed three times with distilled water. Following, seeds were laid between two layers of damp paper at 25°C and left to germinate in the dark for about 3 days. Uniformly germinated seeds with 2–3 cm coleoptiles were selected and sown in pots filled with peat, vermiculite, and perlite (10:1:1 by vol.) Seedlings were grown in an incubator (RXZ-0450, Ningbo Jiangnan Instrument Factory) at 25/20°C (day/night), 450 lmol m^−2^ s^−1^ light density, and a 14/10 h (light/dark) photoperiod for 2 weeks until three leaves had developed. The seedlings were then subjected to the freezing stress experiment, using a treatment of −1°C for 3 h (Shou et al., 2004) followed by 25°C for 3 days. Afterward, seedling survival rates were recorded.

### Physiological analyses of freeze-treated maize seedlings

To analyze the physiological changes of maize seedlings under the freezing treatment, electrical conductivities (both before and after freezing stress) were measured (Griffith and Mclntyre, 1993). Two replicates of 0.1 g fresh leaves were sampled and cut into 2-cm pieces. The leaf samples were then rinsed in pure water to remove cellular proteins from the cut ends and placed in tubes with 10 ml distilled water. After 20 h, the initial electrical conductivity (R1) was measured at room temperature using a METTLER TOLEDO FE30 CONDUCTIVITY (METERMettler Toledo Instruments Co., LTD, Shanghai). The samples were then boiled for 30 min and measured again for the final electrical conductivity (R2). The relative electrical conductivity was calculated as R1/R2.

### Total RNA isolation, cDNA library construction, and sequencing

Maize inbred lines KR701 (freezing tolerant) and Hei8834 (freezing sensitive) were grown according to the method above. For isolation of total RNA, maize seedlings at the three-leaf stage were exposed to a freezing condition (−1°C) for only 1 h (h). The third leaves of both KR701 and Hei8834 were sampled at 0 h (prior to freezing) and after 1 h freezing treatment with liquid nitrogen and then stored in −80°C for further transcriptome analysis. The total RNA of the leaf samples was extracted using Trizol reagent (Invitrogen) and purified using the RNeasy Plant kit (Invitrogen, CA, USA). The RNA quality was checked by a NanoDrop 1000 spectrophotometer and an agarose gel electrophoresis system. The cDNA library construction and sequencing were conducted by BGI Company (Shenzhen, China). In brief, poly-A-containing mRNA was isolated and then subjected to cDNA synthesis. Short mRNA fragments were purified for end reparation and single nucleotide A addition, and then connected with adapters. Next, the cDNA library was obtained by PCR amplification and sequenced on the Illumina Hiseq 2000 platform with 100 cycles of paired-end (2 × 101 bp) sequencing. Raw sequence data were obtained for further analysis. The sequence reads were submitted to GenBank GEO database under accession number SRP065916.

### Processing and mapping of illumina reads

The raw data (raw reads) were filtered with FASTQ_Quality_Filter tool from the FASTX-toolkit. The clean data were used for further analysis.

After preprocessing the RNA-seq data, the reads were mapped to the maize reference genome version 3 (B73 RefGen_v3) (Hubbard et al., 2002) using a spliced aligner Tophat, which was downloaded from http://ftp.maizesequence.org/current/ (Trapnell et al., 2009; Kakumanu et al., 2012).

### Transcript assembly and differential expression analysis

The Sequence Alignment generated by Tophat was then processed by the software Cufflinks (Trapnell et al., 2010) to assemble the alignments in the Sequence Alignment/Map file into transcript fragments (transfrags). FPKM was used as the unit of measurement to estimate transcript abundance. Differential expression analysis of four samples was performed using the Cuffdiff program (Trapnell et al., 2013). *P*-value was adjusted using the *q*-value (Storey, 2003).

### Gene ontology (GO) analysis of selected DEGs

Candidate DEGs were submitted to agriGO (GO analysis toolkit and database for the agriculture community; http://bioinfo.cau.edu.cn/agriGO/index.php) for gene ontology analysis. Enriched GO terms were selected using Singular Enrichment Analysis (SEA) with the maize reference genome B73 as background (Maizesequence, version: 5b). The overrepresented terms in the three categories—biological process, cellular component, and molecular function—were filtered by statistical information, including Fisher's exact test and the Bonferroni multi-test adjustment method (Du et al., 2010; Han et al., 2015).

### Quantitative real-time RT-PCR

Quantitative real-time PCR (qRT-PCR) primers were designed for 30 genes. Gene-specific primers were designed online (https://www.genscript.com/ssl-bin/app/primer) (Table S10). Total RNA was isolated from seedling leaves. For cDNA synthesis, 1 μg of total RNA was reverse-transcribed in a total volume of 25 μL, using One-step gDNA Removal and cDNA Synthesis Super Mix (TRansScript). Synthesized cDNA samples were diluted 10 times prior to qRT-PCR. ZmActin1 was used as the internal reference gene in this study. qRT-PCR was performed with 2 μl of template cDNA, 0.5 μl of forward primer (50 pmol), 0.5 μl of reverse primer (50 pmol), and 10 μl of SYBR Green mix (TOYOBO, Japan) in a total reaction volume of 20 μl. qRT-PCR was carried out in an iQ5 (Bio-Rad) thermocycler. Each sample had three technical replicates.

## Discussion

Along with the climate changes caused by greenhouse gas emissions, freezing and other abiotic stresses are posing ever-growing threats to global food security. Plant responses to these stresses are complicated processes with evolutionary wisdom. Understanding the molecular mechanisms underlying stress responses and applying this knowledge to plant breeding and agricultural practice is of great significance. Transcriptomic analysis using RNA-seq is a powerful tool to monitor global gene expression status and to discover genes responsible for molecular functions and biological processes. Therefore, more and more researchers use RNA-seq analysis to study the abiotic stress response of different species (e.g., An et al., 2012; Raney et al., 2014; Sobkowiak et al., 2014).

Originating in a tropical area, maize is inherently frost-sensitive. However, natural variations in freezing and chilling tolerance exist in different maize populations. As shown with our 30 different inbred lines, seedling survival rate after 3 h of freezing treatment can range from 5.01 to 97.3%. Therefore, investigating the molecular mechanisms that contribute to the freezing response variation in maize is both reasonable and important. Four different groups of RNA-seq data were prepared and analyzed, using two different maize inbred lines (freezing tolerant and freezing sensitive) and two treatments (before and after freezing conditions). By making multiple comparisons of these four groups, we were able to isolate functional information for both the common and differential responsive genes.

In general, plant cells sense low temperatures (chilling or freezing) at the plasma membrane (Pearce, 1999). After signal reception, downstream signaling pathways are activated, including hormone signaling, Ca^2+^ signaling, and ROS signaling (Miura and Furumoto, 2013).

### Hormone signaling in freezing stress response

Previous studies have found that plant hormones participate in low temperature (including chilling and freezing) responses of plants. For example, a number of genes involved in the biosynthesis or signaling of plant hormones, such as abscisic acid, gibberellic acid, and auxin, are regulated by cold stress (Lee, 2005). Several auxin-responsive genes are differentially regulated under various abiotic stress conditions, suggesting that auxin may play an important role in abiotic stress signaling (Jain and Khurana, 2009).

We identified several DEGs involved in auxin signaling such as GRMZM2G344388 (Arabidopsis homolog AT1G18350, also known as ATMKK7 or BUD1, encodes a MAP kinase kinase) and GRMZM2G114850 (Arabidopsis homolog AT1G56010, also known as NAC1, encodes a NAC family transcription factor). Genes involved in the regulation of jasmonic acid, brassinosteroid, cytokinin, and ethylene were also identified, suggesting these plant hormones may also play important roles in freezing stress signaling. Additionally, we identified GRMZM2G044194, which encodes phytosulfokine, suggesting that small peptide signaling may also be an important pathway in freezing stress signaling.

### Ca^2+^-mediated freezing stress signaling

Ca^2+^-mediated signal transduction is one of the most focused signaling pathways in plants and animals. Cold-shock causes a transient rise in cytosolic calcium levels, which probably results (at least in part) from cold-induced opening of plasma membrane calcium channels (Monroy and Dhindsa, 1995; Lewis et al., 1997). In mammals, Ca^2+^ channels can interact with heterotrimeric guanine nucleotide-binding protein (G protein) complexes in response to stress (Wang and Chong, 2010). Recently, Ma et al. (2015) found that the QTL COLD1, which may encode a regulator of G-protein signaling (RGS) with GTPase-accelerating activity, interacts with G protein to activate the Ca^2+^ channel for temperature sensing in rice. Consequently, the expression of stress-responsive genes such as transcription factors, metabolic enzymes, and ion transporters are up-regulated. That is, the up-regulated calcium ion binding protein can activate the downstream cascade kinases, leading to the stimulation of cold-responsive genes. More importantly, their study identified a SNP in COLD1 that may explain the cold tolerance differences between two subspecies of Asian cultivated rice.

In our study, the GO term “G-protein coupled receptor protein signaling pathway” was one of the significantly enriched GO terms in the common freezing responsive genes. For terms associated with molecular function, genes involved in binding (ice binding, DNA binding, and metal ion binding, etc.), catalytic activity (protein tyrosine kinase activity, hydrolase activity, and phosphatase activity, etc.), and receptor activity were significantly enriched. For example, we identified DEG GRMZM2G459663, which encodes a Calcium-binding EF-hand family protein. These data further support the important role of calcium signaling in the freezing stress signaling of plants.

### ROS signaling in freezing stress response

Reactive Oxygen Species (ROS) such as superoxide, hydrogen peroxide, and hydroxyl radicals accumulate under chilling conditions (Suzuki and Mittler, 2006). To prevent the lethal damage caused by ROS, plants have evolved complex antioxidant systems. Several ROS scavenging enzymes have been identified, including catalase (CAT), superoxide dismutase (SOD), and glutathione transferase (GST).

In our RNA-seq data, we also identified genes involved in ROS scavenging. For example, GRMZM2G175728, which encodes a copper chaperone for SOD1, is up-regulated in response to copper and senescence. GRMZM2G075974, which encodes a glutamine amidotransferase, may be involved in defense response. AT5G56550 (Arabidopsis homolog of GRMZM2G076844), also known as OXIDATIVE STRESS 3 (OXS3), is involved in tolerance to heavy metals and oxidative stress.

Ultimately, in order to fully understand the mechanisms of freezing tolerance, plant hormones, Ca^2+^ level, and ROS level should be thoroughly investigated during the cloning process and functional analysis of these potential candidate genes.

### The transcriptomic difference between tolerant and sensitive lines before freezing treatment

From our RNA-seq data, we found that, for the two selected inbred lines, more DEGs existed before freezing treatment compared to after freezing treatment. This finding suggests that the different molecular basis between the tolerant and sensitive lines before freezing treatment are important for their different freezing stress responses. Detailed GO enrichment analysis of CT-upregulated genes and CS-upregulated genes also showed differences in the GO terms enriched in the biological process category. In the tolerant line, GO terms related to metabolic process and protein phosphorylation were enriched. In the sensitive line, GO terms related to transcription, G-protein signaling pathway, and nitrogen compound metabolic process were enriched. Thus, these biological processes may play major roles in the different freezing responses between these two inbred lines. As for the shared GO terms, the genes and the number of genes in each term were different, which may also explain the lines' different freezing sensitivities.

In conclusion, through a freezing-tolerance screening of maize inbred lines from the Heilongjiang Province with the highest latitude in China, we identified two inbred lines with extreme, but opposite, responses—highly tolerant and highly sensitive. Using these two lines and an RNA-seq approach, we obtained candidate genes with different expression patterns and with enrichment on specific gene ontologies. The top 30 candidate genes were repeatedly confirmed by using QRT-PCR, which resulted in high correlation with our RNA-seq-based findings. This study provided valuable resources for further studies to enhance understanding of the molecular mechanisms underlying the freezing response of maize. Ultimately, a greater understanding of these molecular mechanisms will enable targeted breeding strategies for developing superior maize varieties able to resist frost and to achieve their full yield potential.

## Author contributions

Conceived and designed the experiments: ZZ, DY, and TW. Performed the experiments: ZL, GH, XL, QZ. Analyzed the data: ZL, GH, YZ, XZ, XY, and YL. Wrote the paper: ZL, GH, and ZZ. Revised the paper: ZZ. All authors read and approved the final manuscript.

### Conflict of interest statement

The authors declare that the research was conducted in the absence of any commercial or financial relationships that could be construed as a potential conflict of interest.

## Supplementary material

### Availability of supporting data

Illumina sequence data are available from NCBI under Short Read Archive accession SRP065916.
