# Supplementary material for: Transcriptome Sequencing Identified Genes and Gene Ontologies Associated with Early Freezing Tolerance in Maize
Source: Front Plant Sci. 2016 Oct 7;7:1477. doi: 10.3389/fpls.2016.01477 (PMC5054024; doi:10.3389/fpls.2016.01477)
Supplement: Supplementary file 2 [file Image2.PDF]

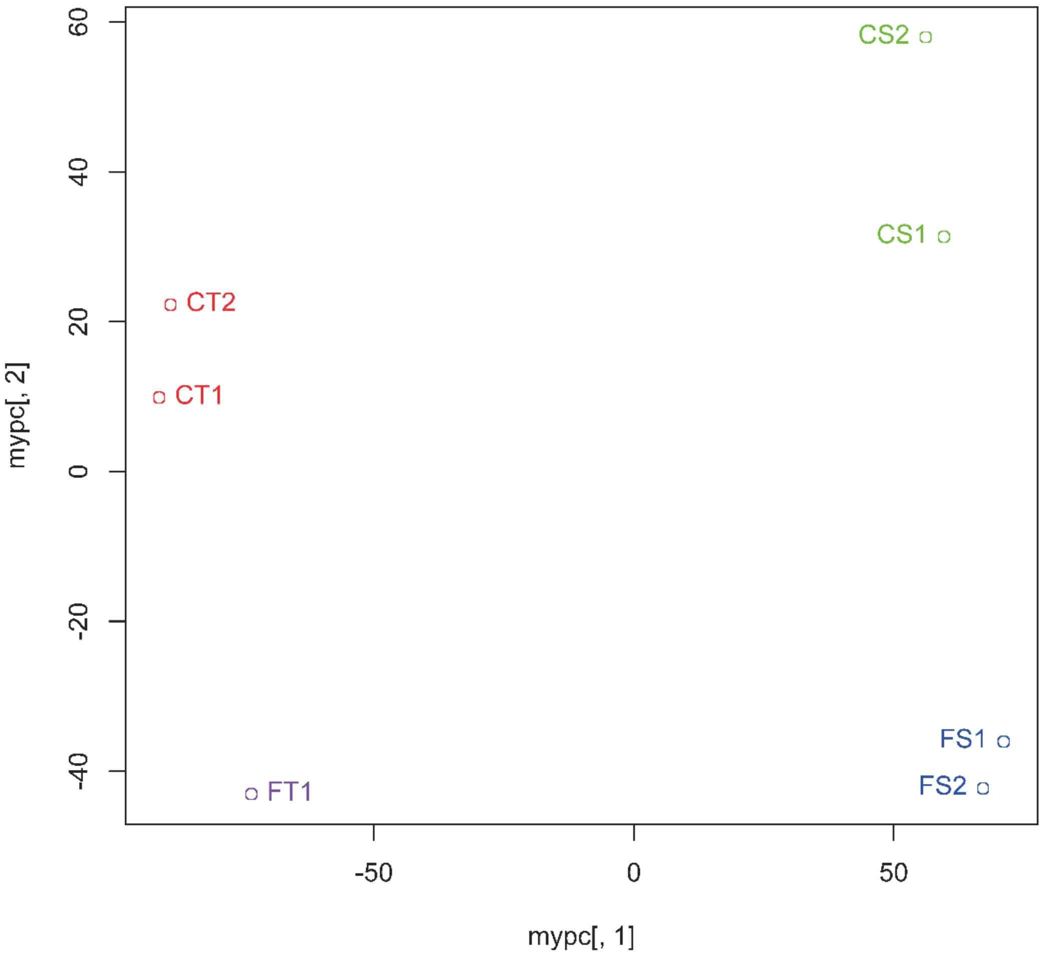

Figure S2 PCA analysis of all samples.

PCA (Principal Component Analysis) of all samples were performed with R version 3.3.0; FT: Tolerant line after freezing; CT: Tolerant line before freezing; FS: Sensitive line after freezing; CS: Sensitive line before freezing. The numbers represent independent replicate.
