# Supplementary material for: Transcriptome Sequencing Identified Genes and Gene Ontologies Associated with Early Freezing Tolerance in Maize
Source: Front Plant Sci. 2016 Oct 7;7:1477. doi: 10.3389/fpls.2016.01477 (PMC5054024; doi:10.3389/fpls.2016.01477)
Supplement: Supplementary file 3 [file Table1.DOCX]

Table S1 The seedling survival rate of 30 Maize inbred lines

| Name of Inbred Line | Seedling survival rate (%) | Name of Inbred Line | Seedling survival rate (%) |
| --- | --- | --- | --- |
| Hei8834 | 5.01 ± 0.20 | Wa40 | 45.60 ± 1.55 |
| Chang7-2 | 16.28 ± 1.14 | HR210 | 46.31 ± 2.84 |
| Zheng58K | 21.86 ± 2.25 | m8 | 53.13 ± 2.56 |
| Mo17G | 21.94 ± 2.60 | He344 | 54.52 ± 1.63 |
| K10FR | 26.20 ± 3.78 | KR774 | 55.08 ± 1.87 |
| Shen125 | 27.27 ± 2.14 | US3-3-2 | 58.76 ± 2.89 |
| DK257 | 29.49 ± 1.27 | HR78 | 59.46 ± 1.40 |
| 335M | 30.71 ± 2.84 | MO17Za | 63.98 ± 2.39 |
| M5911 | 30.86 ± 1.25 | 335F | 65.74 ± 2.29 |
| DK277 | 35.63 ± 2.34 | Nan9808 | 68.92 ± 1.76 |
| 3004 | 39.87 ± 2.31 | DK429 | 71.34 ± 1.45 |
| KL3 | 41.60 ± 2.19 | HR02 | 71.44 ± 2.22 |
| KF27 | 44.34 ± 2.06 | 811 | 74.63 ± 3.75 |
| k10 | 45.38 ± 2.86 | B125 | 83.94 ± 2.48 |
| HR73 | 45.57 ± 2.09 | KR701 | 95.43 ± 1.46 |
