# Supplementary material for: Transcriptome Sequencing Identified Genes and Gene Ontologies Associated with Early Freezing Tolerance in Maize
Source: Front Plant Sci. 2016 Oct 7;7:1477. doi: 10.3389/fpls.2016.01477 (PMC5054024; doi:10.3389/fpls.2016.01477)
Supplement: Supplementary file 4 [file Table2.DOCX]

**Table S2** Summary of trimming and alignment

| Samples | Clean reads | Read length(bp) | Q20(%) | | GC(%) | Mapped reads  (%, mapped/cleaned) |
| --- | --- | --- | --- | --- | --- | --- |
| CT_R1 | 20293016 | 100 | 98.42% | 52.17% | | 83.5% |
| CT_R2 | 25836442 | 100 | 98.50% | 52.04% | | 82.7% |
| CS_R1 | 22476004 | 100 | 98.49% | 52.23% | | 76.1% |
| CS_R2 | 22263446 | 100 | 98.36% | 53.51% | | 74.5% |
| FT_R1 | 22952424 | 100 | 98.40% | 53.48% | | 83.6% |
| FS_R1 | 19019500 | 100 | 98.41% | 52.90% | | 74.9% |
| FS_R2 | 21647634 | 100 | 98.45% | 51.85% | | 75.6% |
| Total | 154488466 |  |  |  | | 78.84% |
