# Supplementary material for: Transcriptome Sequencing Identified Genes and Gene Ontologies Associated with Early Freezing Tolerance in Maize
Source: Front Plant Sci. 2016 Oct 7;7:1477. doi: 10.3389/fpls.2016.01477 (PMC5054024; doi:10.3389/fpls.2016.01477)
Supplement: Supplementary file 5 [file Table3.DOCX]

| Treatment | Number of samples | | Minimum genes expressed | Maximum genes expressed | Total number of genes expressed |
| --- | --- | --- | --- | --- | --- |
| CT | | 2 | 16,400 | 16,460 | 16,642 |
| CS | | 2 | 16,830 | 17,236 | 17,292 |
| FT | | 1 | 16,761 | 16,761 | 16,761 |
| FS | | 2 | 17,071 | 17,236 | 17,352 |
| Total | |  |  |  | 19,684 |

Table S3 Number of genes expressed in four different treatments
