# Supplementary material for: Transcriptome Sequencing Identified Genes and Gene Ontologies Associated with Early Freezing Tolerance in Maize
Source: Front Plant Sci. 2016 Oct 7;7:1477. doi: 10.3389/fpls.2016.01477 (PMC5054024; doi:10.3389/fpls.2016.01477)
Supplement: Supplementary file 6 [file Table4.DOCX]

Table S4 GO analysis of genes specifically expressed in tolerant line KR701 after freezing treatment (FT)

| **GO term** | **Ontology** | **Description** | **Gene Number** | **p-value** | **FDR** |
| --- | --- | --- | --- | --- | --- |
| GO:0007186 | P | G-protein coupled receptor protein signaling pathway | [7](http://bioinfo.cau.edu.cn/agriGO/termDetail.php?session=740989588&GO=GO:0007186) | 2.50E-07 | 0.0001 |
| GO:0008152 | P | metabolic process | [78](http://bioinfo.cau.edu.cn/agriGO/termDetail.php?session=740989588&GO=GO:0008152) | 0.00017 | 0.036 |
| GO:0032555 | F | purine ribonucleotide binding | [36](http://bioinfo.cau.edu.cn/agriGO/termDetail.php?session=740989588&GO=GO:0032555) | 1.70E-11 | 2.20E-09 |
| GO:0005488 | F | binding | [98](http://bioinfo.cau.edu.cn/agriGO/termDetail.php?session=740989588&GO=GO:0005488) | 1.80E-10 | 1.20E-08 |
| GO:0017076 | F | purine nucleotide binding | [36](http://bioinfo.cau.edu.cn/agriGO/termDetail.php?session=740989588&GO=GO:0017076) | 1.60E-10 | 1.20E-08 |
| GO:0004713 | F | protein tyrosine kinase activity | [12](http://bioinfo.cau.edu.cn/agriGO/termDetail.php?session=740989588&GO=GO:0004713) | 1.20E-09 | 4.60E-08 |
| GO:0005524 | F | ATP binding | [30](http://bioinfo.cau.edu.cn/agriGO/termDetail.php?session=740989588&GO=GO:0005524) | 1.00E-09 | 4.60E-08 |
| GO:0003824 | F | catalytic activity | [78](http://bioinfo.cau.edu.cn/agriGO/termDetail.php?session=740989588&GO=GO:0003824) | 1.10E-06 | 2.30E-05 |
| GO:0046914 | F | transition metal ion binding | [28](http://bioinfo.cau.edu.cn/agriGO/termDetail.php?session=740989588&GO=GO:0046914) | 5.00E-06 | 0.0001 |
| GO:0009055 | F | electron carrier activity | [10](http://bioinfo.cau.edu.cn/agriGO/termDetail.php?session=740989588&GO=GO:0009055) | 8.00E-05 | 0.0015 |
| GO:0008509 | F | anion transmembrane transporter activity | [5](http://bioinfo.cau.edu.cn/agriGO/termDetail.php?session=740989588&GO=GO:0008509) | 8.60E-05 | 0.0015 |
| GO:0046872 | F | metal ion binding | [31](http://bioinfo.cau.edu.cn/agriGO/termDetail.php?session=740989588&GO=GO:0046872) | 0.0001 | 0.0017 |
| GO:0043169 | F | cation binding | [31](http://bioinfo.cau.edu.cn/agriGO/termDetail.php?session=740989588&GO=GO:0043169) | 0.00022 | 0.0034 |
| GO:0008270 | F | zinc ion binding | [17](http://bioinfo.cau.edu.cn/agriGO/termDetail.php?session=740989588&GO=GO:0008270) | 0.00069 | 0.0092 |
| GO:0016740 | F | transferase activity | [32](http://bioinfo.cau.edu.cn/agriGO/termDetail.php?session=740989588&GO=GO:0016740) | 0.0007 | 0.0092 |
| GO:0004871 | F | signal transducer activity | [8](http://bioinfo.cau.edu.cn/agriGO/termDetail.php?session=740989588&GO=GO:0004871) | 0.00087 | 0.01 |
| GO:0004674 | F | protein serine/threonine kinase activity | [12](http://bioinfo.cau.edu.cn/agriGO/termDetail.php?session=740989588&GO=GO:0004674) | 0.0013 | 0.014 |
| GO:0020037 | F | heme binding | [7](http://bioinfo.cau.edu.cn/agriGO/termDetail.php?session=740989588&GO=GO:0020037) | 0.0019 | 0.019 |
| GO:0070011 | F | peptidase activity, acting on L-amino acid peptides | [8](http://bioinfo.cau.edu.cn/agriGO/termDetail.php?session=740989588&GO=GO:0070011) | 0.0019 | 0.019 |
| GO:0005525 | F | GTP binding | [7](http://bioinfo.cau.edu.cn/agriGO/termDetail.php?session=740989588&GO=GO:0005525) | 0.0028 | 0.024 |
| GO:0005515 | F | protein binding | [25](http://bioinfo.cau.edu.cn/agriGO/termDetail.php?session=740989588&GO=GO:0005515) | 0.0026 | 0.024 |
| GO:0046906 | F | tetrapyrrole binding | [7](http://bioinfo.cau.edu.cn/agriGO/termDetail.php?session=740989588&GO=GO:0046906) | 0.0032 | 0.026 |
| GO:0004872 | F | receptor activity | [6](http://bioinfo.cau.edu.cn/agriGO/termDetail.php?session=740989588&GO=GO:0004872) | 0.0031 | 0.026 |
| GO:0022892 | F | substrate-specific transporter activity | [11](http://bioinfo.cau.edu.cn/agriGO/termDetail.php?session=740989588&GO=GO:0022892) | 0.0043 | 0.033 |
| GO:0008233 | F | peptidase activity | [8](http://bioinfo.cau.edu.cn/agriGO/termDetail.php?session=740989588&GO=GO:0008233) | 0.0045 | 0.034 |
| GO:0005506 | F | iron ion binding | [9](http://bioinfo.cau.edu.cn/agriGO/termDetail.php?session=740989588&GO=GO:0005506) | 0.0049 | 0.036 |

**P: biological process; F: molecular function**
