# Supplementary material for: Transcriptome Sequencing Identified Genes and Gene Ontologies Associated with Early Freezing Tolerance in Maize
Source: Front Plant Sci. 2016 Oct 7;7:1477. doi: 10.3389/fpls.2016.01477 (PMC5054024; doi:10.3389/fpls.2016.01477)
Supplement: Supplementary file 7 [file Table5.DOCX]

Table S5 GO analysis of genes specifically expressed in sensitive line Hei8834 after freezing treatment (FS)

| GO term | Ontology | Description | Gene Number | p-value | FDR |
| --- | --- | --- | --- | --- | --- |
| GO:0050826 | P | response to freezing | 14 | 2.40E-16 | 9.90E-14 |
| GO:0045449 | P | regulation of transcription | 40 | 4.90E-11 | 6.60E-09 |
| GO:0019219 | P | regulation of nucleobase, nucleoside, nucleotide and nucleic acid metabolic process | 40 | 2.10E-10 | 1.70E-08 |
| GO:0031326 | P | regulation of cellular biosynthetic process | 41 | 5.20E-10 | 2.40E-08 |
| GO:0008152 | P | metabolic process | 133 | 4.30E-10 | 2.40E-08 |
| GO:0009409 | P | response to cold | 14 | 1.00E-09 | 4.10E-08 |
| GO:0043170 | P | macromolecule metabolic process | 100 | 1.10E-09 | 4.20E-08 |
| GO:0019222 | P | regulation of metabolic process | 42 | 1.20E-08 | 3.00E-07 |
| GO:0006468 | P | protein amino acid phosphorylation | 25 | 1.30E-08 | 3.00E-07 |
| GO:0007186 | P | G-protein coupled receptor protein signaling pathway | 9 | 2.70E-08 | 5.60E-07 |
| GO:0042592 | P | homeostatic process | 15 | 6.40E-08 | 1.20E-06 |
| GO:0065007 | P | biological regulation | 58 | 8.10E-08 | 1.50E-06 |
| GO:0009266 | P | response to temperature stimulus | 14 | 1.30E-07 | 2.40E-06 |
| GO:0006139 | P | nucleobase, nucleoside, nucleotide and nucleic acid metabolic process | 52 | 3.90E-07 | 6.40E-06 |
| GO:0006796 | P | phosphate metabolic process | 30 | 1.40E-06 | 2.00E-05 |
| GO:0050794 | P | regulation of cellular process | 48 | 3.50E-06 | 4.30E-05 |
| GO:0046942 | P | carboxylic acid transport | 6 | 6.70E-06 | 7.60E-05 |
| GO:0032774 | P | RNA biosynthetic process | 21 | 0.00016 | 0.0015 |
| GO:0007166 | P | cell surface receptor linked signaling pathway | 9 | 0.00022 | 0.0021 |
| GO:0009628 | P | response to abiotic stimulus | 14 | 0.0052 | 0.042 |
| GO:0004713 | F | protein tyrosine kinase activity | 25 | 4.50E-21 | 1.70E-18 |
| GO:0005488 | F | binding | 154 | 1.10E-16 | 2.10E-14 |
| GO:0032555 | F | purine ribonucleotide binding | 44 | 3.60E-10 | 3.40E-08 |
| GO:0005524 | F | ATP binding | 39 | 7.40E-10 | 5.60E-08 |
| GO:0030528 | F | transcription regulator activity | 28 | 3.00E-09 | 1.30E-07 |
| GO:0003824 | F | catalytic activity | 118 | 8.90E-09 | 2.80E-07 |
| GO:0004674 | F | protein serine/threonine kinase activity | 25 | 1.90E-08 | 5.50E-07 |
| GO:0050825 | F | ice binding | 14 | 4.50E-07 | 8.50E-06 |
| GO:0005199 | F | structural constituent of cell wall | 6 | 4.80E-07 | 8.50E-06 |
| GO:0043565 | F | sequence-specific DNA binding | 13 | 1.70E-06 | 2.80E-05 |
| GO:0070001 | F | aspartic-type peptidase activity | 6 | 2.40E-06 | 3.60E-05 |
| GO:0016772 | F | transferase activity, transferring phosphorus-containing groups | 35 | 2.90E-06 | 4.10E-05 |
| GO:0060089 | F | molecular transducer activity | 12 | 7.00E-05 | 0.00087 |
| GO:0046943 | F | carboxylic acid transmembrane transporter activity | 5 | 0.00012 | 0.0015 |
| GO:0009055 | F | electron carrier activity | 12 | 0.00018 | 0.002 |
| GO:0046914 | F | transition metal ion binding | 33 | 0.00021 | 0.0023 |
| GO:0008509 | F | anion transmembrane transporter activity | 5 | 0.00062 | 0.0064 |
| GO:0046872 | F | metal ion binding | 37 | 0.0036 | 0.036 |
| GO:0016787 | F | hydrolase activity | 35 | 0.0046 | 0.042 |
| GO:0015291 | F | secondary active transmembrane transporter activity | 6 | 0.0044 | 0.042 |
| GO:0000151 | C | ubiquitin ligase complex | 5 | 0.00021 | 0.021 |

**P: biological process; F: molecular function; C:** **cellular component**
