# Supplementary material for: Transcriptome Sequencing Identified Genes and Gene Ontologies Associated with Early Freezing Tolerance in Maize
Source: Front Plant Sci. 2016 Oct 7;7:1477. doi: 10.3389/fpls.2016.01477 (PMC5054024; doi:10.3389/fpls.2016.01477)
Supplement: Supplementary file 8 [file Table6.DOCX]

Table S6 GO analysis of freezing responsive genes shared by the tolerant and sensitive lines

| GO term | Ontology | Description | Gene Number | p-value | FDR |
| --- | --- | --- | --- | --- | --- |
| GO:0005488 | F | binding | 48 | 5.50E-06 | 0.00072 |
| GO:0004713 | F | protein tyrosine kinase activity | 6 | 1.20E-05 | 0.00081 |
| GO:0003677 | F | DNA binding | 13 | 0.00037 | 0.016 |
| GO:0030528 | F | transcription regulator activity | 9 | 0.00054 | 0.017 |
| GO:0032555 | F | purine ribonucleotide binding | 13 | 0.00097 | 0.021 |
| GO:0005524 | F | ATP binding | 11 | 0.0021 | 0.033 |
| GO:0004674 | F | protein serine/threonine kinase activity | 7 | 0.0046 | 0.044 |
| GO:0070011 | F | peptidase activity, acting on L-amino acid peptides | 5 | 0.0046 | 0.044 |
| GO:0046872 | F | metal ion binding | 15 | 0.006 | 0.046 |
| GO:0046914 | F | transition metal ion binding | 12 | 0.0067 | 0.046 |
| GO:0008270 | F | zinc ion binding | 9 | 0.0068 | 0.046 |

**F: molecular function**
