# Supplementary material for: Transcriptome Sequencing Identified Genes and Gene Ontologies Associated with Early Freezing Tolerance in Maize
Source: Front Plant Sci. 2016 Oct 7;7:1477. doi: 10.3389/fpls.2016.01477 (PMC5054024; doi:10.3389/fpls.2016.01477)
Supplement: Supplementary file 9 [file Table7.DOCX]

Table S7 Detailed information for 151 up-up regulated genes during freezing stress

| **GO term** | **Ontology** | **Description** | **Number** | **p-value** | **FDR** |
| --- | --- | --- | --- | --- | --- |
| GO:0050826 | P | response to freezing | 10 | 1.80E-14 | 5.10E-12 |
| GO:0007186 | P | G-protein coupled receptor protein signaling pathway | 8 | 6.40E-10 | 8.90E-08 |
| GO:0042592 | P | homeostatic process | 11 | 1.90E-08 | 1.40E-06 |
| GO:0045449 | P | regulation of transcription | 19 | 1.50E-06 | 6.80E-05 |
| GO:0065007 | P | biological regulation | 30 | 3.30E-06 | 9.80E-05 |
| GO:0051171 | P | regulation of nitrogen compound metabolic process | 19 | 3.10E-06 | 9.80E-05 |
| GO:0031326 | P | regulation of cellular biosynthetic process | 19 | 8.80E-06 | 0.00017 |
| GO:0065008 | P | regulation of biological quality | 11 | 8.50E-06 | 0.00017 |
| GO:0060255 | P | regulation of macromolecule metabolic process | 19 | 4.40E-05 | 0.00066 |
| GO:0009628 | P | response to abiotic stimulus | 11 | 0.00012 | 0.0017 |
| GO:0050794 | P | regulation of cellular process | 22 | 0.00078 | 0.0099 |
| GO:0032501 | P | multicellular organismal process | 10 | 0.0028 | 0.031 |
| GO:0043687 | P | post-translational protein modification | 12 | 0.0036 | 0.039 |
| GO:0005488 | F | binding | 68 | 2.90E-08 | 6.80E-06 |
| GO:0050825 | F | ice binding | 10 | 2.30E-07 | 1.80E-05 |
| GO:0003677 | F | DNA binding | 21 | 6.50E-07 | 3.80E-05 |
| GO:0008509 | F | anion transmembrane transporter activity | 5 | 1.40E-05 | 0.00065 |
| GO:0004713 | F | protein tyrosine kinase activity | 6 | 8.20E-05 | 0.0032 |
| GO:0030528 | F | transcription regulator activity | 12 | 0.00011 | 0.0036 |
| GO:0046872 | F | metal ion binding | 23 | 0.0002 | 0.0052 |
| GO:0003824 | F | catalytic activity | 50 | 0.00049 | 0.0095 |
| GO:0016787 | F | hydrolase activity | 20 | 0.0015 | 0.026 |
| GO:0016788 | F | hydrolase activity, acting on ester bonds | 8 | 0.0024 | 0.031 |
| GO:0016791 | F | phosphatase activity | 5 | 0.002 | 0.031 |
| GO:0004871 | F | signal transducer activity | 6 | 0.0023 | 0.031 |
| GO:0004872 | F | receptor activity | 5 | 0.0028 | 0.035 |
| GO:0016747 | F | transferase activity, transferring acyl groups other than amino-acyl groups | 5 | 0.0034 | 0.04 |
| GO:0005524 | F | ATP binding | 13 | 0.004 | 0.043 |
| GO:0042578 | F | phosphoric ester hydrolase activity | 5 | 0.004 | 0.043 |
| GO:0016757 | F | transferase activity, transferring glycosyl groups | 6 | 0.005 | 0.049 |
| GO:0016021 | C | integral to membrane | 16 | 2.50E-05 | 0.0007 |

**P: biological process; F: molecular function; C:** **cellular component**
