# Supplementary material for: Transcriptome Sequencing Identified Genes and Gene Ontologies Associated with Early Freezing Tolerance in Maize
Source: Front Plant Sci. 2016 Oct 7;7:1477. doi: 10.3389/fpls.2016.01477 (PMC5054024; doi:10.3389/fpls.2016.01477)
Supplement: Supplementary file 10 [file Table8.DOCX]

Table S8 Arabidopsis homologue search results for common response genes (UU and DD) with significant differential responsive levels and for different response genes (UD and DU) between the freezing tolerant and freezing sensitive maize lines.

| **Name** | **CT_FT log2 fold change** | **p-value** | **CS_FS log2 fold change** | **p-value** | **Best hit in Arabidopsis** | **GO Description** |
| --- | --- | --- | --- | --- | --- | --- |
| GRMZM2G148333 | 1.22522 | 0.00105 | 0.874917 | 0.00565 | AT1G72360.2 | DNA binding |
| GRMZM2G344388 | 1.41399 | 0.0016 | -1.14814 | 0.0001 | AT1G18350.1 | nucleotide binding |
| GRMZM2G097135 | -3.20384 | 0.00405 | 1.72991 | 0.00005 | AT1G12060.1 | chaperone binding |
| GRMZM2G171311 | -1.08773 | 0.0012 | 1.31663 | 0.00005 | AT5G65630.1 | protein binding |
| GRMZM2G101020 | -1.35018 | 0.6542 | 0.892227 | 0.5218 | AT1G19910.1 | vacuole |
| GRMZM2G086841 | 3.15597 | 0.0039 | 1.00881 | 0.0002 | AT1G80920.1 | response to light stimulus |
| GRMZM2G125775 | 3.0845 | 0.0055 | 1.17464 | 0.0009 | AT3G28210.1 | zinc ion binding |
| GRMZM2G076844 | 1.99317 | 0.0004 | 0.851083 | 0.0059 | AT5G56550.1 | - |
| GRMZM2G000236 | 5.10287 | 0.0043 | 2.30387 | 0.00005 | AT1G76680.1 | catalytic activity |
| GRMZM2G165192 | 2.36877 | 0.00005 | 1.07875 | 0.00005 | AT2G39980.1 | transferase activity, transferring acyl groups other than amino-acyl groups |
| GRMZM2G057823 | 1.52742 | 0.00005 | 0.755496 | 0.0053 | AT2G36460.1 | catalytic activity |
| GRMZM2G149422 | 4.6206 | 0.0001 | 2.29598 | 0.0001 | AT4G08950.1 | - |
| GRMZM2G420715 | 3.16001 | 0.0001 | 1.57178 | 0.0002 | AT4G39720.1 | - |

UU: genes up-regulated in both CT_FT and CS_FS; DD: genes down-regulated in both CT_FT and CS_FS; UD: genes up-regulated in CT_FT, but down-regulated in CS_FS; DU: genes down-regulated in CT_FT, but up-regulated in CS_FS.
