# Supplementary material for: Transcriptome Sequencing Identified Genes and Gene Ontologies Associated with Early Freezing Tolerance in Maize
Source: Front Plant Sci. 2016 Oct 7;7:1477. doi: 10.3389/fpls.2016.01477 (PMC5054024; doi:10.3389/fpls.2016.01477)
Supplement: Supplementary file 11 [file Table9.DOCX]

Table S9 DEGs in area II (Figure 3) that were assigned “response to freezing” in the GO analysis

| **Name** | **log2**  **(FT/FS)** | **p-value** | **Description** | **Best hit in Arabidopsis** | **Description** |
| --- | --- | --- | --- | --- | --- |
| GRMZM2G075974 | 1.78 | 0.0068 | Glutamine amidotransferase superfamily | AT2G23970.1 | Class I glutamine amidotransferase-like superfamily protein |
| GRMZM2G103812 | 1.47 | 0.0001 | Selenium-binding protein | AT4G14040.1 | selenium-binding protein 2 |
| GRMZM2G115422 | 1.00 | 0.00315 | Transferase | AT2G39980.1 | HXXXD-type acyl-transferase family protein |
| GRMZM2G459663 | 2.29 | 0.0039 | Calcium-binding EF-hand | AT5G39670.1 | Calcium-binding EF-hand family protein |
| GRMZM2G079956 | 1.60 | 0.0048 | Apoptosis regulator, Bcl-2 protein, BAG | AT5G07220.1 | BCL-2-associated athanogene 3 |
| GRMZM2G061932 | 1.09 | 0.00365 | Antifreeze protein, type I | AT2G41475.1 | Embryo-specific protein 3, (ATS3) |
| GRMZM2G369839 | 1.28 | 0.00175 | Orphan nuclear receptor, NOR1 type | 0 | 0 |
| GRMZM2G348452 | 1.68 | 0.0004 | Cytokinin dehydrogenase 1, FAD/cytokinin binding domain | AT5G21482.1 | cytokinin oxidase 7 |
| GRMZM2G350662 | 9.34 | 0.00005 | Antifreeze protein, type I | 0 | 0 |
| GRMZM2G175728 | -1.47 | 0.00595 | Heavy metal transport/detoxification protein | AT1G12520.1 | copper chaperone for SOD1 |
| AC187243.3_FG005 | -7.90 | 0.00065 | Antifreeze protein, type I | 0 | 0 |
| GRMZM2G044194 | -7.23 | 0.00065 | Phytosulfokine | AT3G49780.1 | phytosulfokine 4 precursor |
| GRMZM2G114850 | -1.68 | 0.00475 | No apical meristem (NAM) protein | AT1G56010.2 | NAC domain containing protein 1 |
| GRMZM2G180335 | -1.08 | 0.00525 | Dynamin GTPase effector | AT4G33650.1 | dynamin-related protein 3A |
| GRMZM2G101142 | -1.98 | 0.00525 | Strictosidine synthase, conserved region | AT3G51420.1 | strictosidine synthase-like 4 |
| GRMZM2G476685 | -6.85 | 0.00005 | Cyclin, C-terminal | AT1G70210.1 | CYCLIN D1;1 |
| GRMZM2G178038 | -1.06 | 0.00005 | Zinc finger, RING-CH-type | AT5G60710.1 | Zinc finger (C3HC4-type RING finger) family protein |
