# Supplementary material for: Transcriptome Sequencing Identified Genes and Gene Ontologies Associated with Early Freezing Tolerance in Maize
Source: Front Plant Sci. 2016 Oct 7;7:1477. doi: 10.3389/fpls.2016.01477 (PMC5054024; doi:10.3389/fpls.2016.01477)
Supplement: Supplementary file 12 [file Table10.DOCX]

Table S10 Primer sequence of identified genes by qRT-PCR

| Gene short name | Forward primer sequence (5’-3’) | Reversed primer sequence (5’-3’) |
| --- | --- | --- |
| ZmGRMZM2G148333 | TAAGGTCGTTCGTGTGGAGC | ACAAGAGCCATACGAGTCGC |
| ZmGRMZM2G344388 | CAGGCGAGTGTGGATATGCA | CAATGACACGGGAAGGAGCT |
| ZmGRMZM2G097135 | GCCAGAAGCGGTGAAGGAAT | CAGTTGTAGGCTCCGCTTCA |
| ZmGRMZM2G171311 | GTCGGTACTTGGGTGATGGG | GCCGGCATTGCTACTTTTCC |
| ZmGRMZM2G101020 | CTATGCCCACCTGTCGTCTG | TGCCAACAAACAACTTCGGC |
| ZmGRMZM2G086841 | CAGCCGGTCGATGGATAAGG | AAAACTAGAGTGGGTGCCGC |
| ZmGRMZM2G125775 | ACAACCCAATAAACCGCGGA | CAGTCGAAGGGCAGGAAGTC |
| ZmGRMZM2G076844 | GGTGATCAGGTTCGAGGAGC | CTCGAAGTGACAGCTGGACC |
| ZmGRMZM2G000236 | CTAGCAAGTGACCGACCGAG | ACTGCCCCATCTTGTAAGGC |
| ZmGRMZM2G165192 | TGAGCGCGTCTTTCACTTCA | CTTGGGGATTCTTGGGGTCC |
| ZmGRMZM2G057823 | GGTGCCAATTGTTGAGCCTG | GTTGGGCTTCAGGAGGGTAC |
| ZmGRMZM2G149422 | GCTCCAAGGGAAGGCTTCAC | CGAGTGAGAGTGCAGGAGTG |
| ZmGRMZM2G420715 | ACACCAGCTCTCATGGCTTC | AGGCTGCTAATCCTCACTCCA |
| ZmGRMZM2G075974 | TCTGGCTGTGCACAAAGACA | ACAAGAGCAACCCAAACCGA |
| ZmGRMZM2G103812 | GGACACTGGCTATGTTGGCT | ATCCAGTTGCGGACCTTCAG |
| ZmGRMZM2G115422 | CCAACAAGTTCGACGGCAAG | CCCGACACGAACTGCATGAA |
| ZmGRMZM2G459663 | CGGATGAGCCTGTTCCAGTT | CTGTCTCATCTCCTGCCACG |
| ZmGRMZM2G079956 | CAAGAAGCTGCTGTCGGAGA | GGTCCTCGAGCATGACCATC |
| ZmGRMZM2G061932 | ATCTCAACGCTGCTCACCTC | TGACTCCGAGACTCCGAGTG |
| ZmGRMZM2G369839 | GCCGCCGCCTAGATCTATTT | CCTGCACCAAGGAAACCAGA |
| ZmGRMZM2G348452 | CTCAACCTCTTCGTCTCCGC | GGGTCCCACTTGCTCTTGAG |
| ZmGRMZM2G350662 | CGTCGAAAAGCAGTCTCCCA | TTCATCTCCATGGCTGCTGG |
| ZmGRMZM2G175728 | AAGCCCATTTCTCAGGACCG | TAGTTCTCTCCTACCCCGGC |
| ZmAC187243.3_FG005 | TTCAGCAAACCAGTCGCGG | CAGAGAGGAAGAAGCCGCC |
| ZmGRMZM2G044194 | ACATCTACACCCAGCAGCAC | ACACACACACACACACGAGT |
| ZmGRMZM2G114850 | ACAACAACACTGCCAGCTCT | GCATCGCGTGGTCTAGGTAG |
| ZmGRMZM2G180335 | AGAGGCAGTAGAGAGGGGTG | CTTGACTGCTTCGGGGTTGA |
| ZmGRMZM2G101142 | ACAAGGGGCTTTTGAGGGTC | TGCTGGCGTCACTGAAGTAG |
| ZmGRMZM2G476685 | GAGGGCGTCAGAGAGAGAGA | CAGAGCACCACCACAGTAGG |
| ZmGRMZM2G178038 | TTCCTTCTCCCCGCCATAGA | CCAGTGCAACGAGAGGAGAG |
| ZmActin-1 | GATGATGCGCCAAGAGCTG | GCCTCATCACCTACGTAGGCAT |
